# Synthesis, Antiproliferative and Antifungal Activities of 1,2,3-Triazole-Substituted Carnosic Acid and Carnosol Derivatives

**DOI:** 10.3390/molecules20058666

**Published:** 2015-05-14

**Authors:** Mariano Walter Pertino, Cristina Theoduloz, Estefania Butassi, Susana Zacchino, Guillermo Schmeda-Hirschmann

**Affiliations:** 1 Instituto de Química de Recursos Naturales, Universidad de Talca, Casilla 747, Talca, Chile; E-Mail: schmeda@utalca.cl; 2 Facultad de Ciencias de la Salud, Universidad de Talca, Casilla 747, Talca, Chile; E-Mail: ctheodul@utalca.cl; 3 Facultad de Ciencias Bioquímicas y Farmacéuticas, Farmacognosia, Universidad Nacional de Rosario, Suipacha 531, Rosario 2000, Argentina; E-Mails: ebutassi@fbioyf.unr.edu.ar (E.B.); szaabgil@citynet.net.ar (S.Z.)

**Keywords:** carnosic acid, carnosol, click chemistry, antiproliferative, antifungal

## Abstract

Abietane diterpenes exhibit an array of interesting biological activities, which have generated significant interest among the pharmacological community. Starting from the abietane diterpenes carnosic acid and carnosol, twenty four new triazole derivatives were synthesized using click chemistry. The compounds differ in the length of the linker and the substituent on the triazole moiety. The compounds were assessed as antiproliferative and antifungal agents. The antiproliferative activity was determined on normal lung fibroblasts (MRC-5), gastric epithelial adenocarcinoma (AGS), lung cancer (SK-MES-1) and bladder carcinoma (J82) cells while the antifungal activity was assessed against *Candida albicans* ATCC 10231 and *Cryptococcus neoformans* ATCC 32264. The carnosic acid γ-lactone derivatives **1**–**3** were the most active antiproliferative compounds of the series, with IC_50_ values in the range of 43.4–46.9 μM and 39.2–48.9 μM for MRC-5 and AGS cells, respectively. Regarding antifungal activity, *C. neoformans* was the most sensitive fungus, with nine compounds inhibiting more than 50% of its fungal growth at concentrations ≤250 µg∙mL^−1^. Compound **22**, possessing a *p*-Br-benzyl substituent on the triazole ring, showed the best activity (91% growth inhibition) at 250 µg∙mL^−1^ In turn, six compounds inhibited 50% *C. albicans* growth at concentrations lower than 250 µg∙mL^−1^.

## 1. Introduction

Metabolites isolated from natural sources, mainly from plants, remain a major source of compounds with pharmacological properties that can be modified to generate new drugs with better effects and lower toxicity [1]. Among the terpenes investigated for pharmacological properties, the abietane diterpenes are a promising group due to their abundance in Nature and occurrence in medicinal plants and industrial wastes [2]. A review of the biological activities of natural and synthetic abietane diterpenes has been published recently [3]. It has been reported that some abietane terpenes are cytotoxic and antiproliferative, leading to new studies with the aim to identify the mechanisms of action of these molecules. A recent review that focused on molecular targets of these terpenes in cancer cells, pointed out the potential of abietanes from *Salvia* as pro-apoptotic agents [4]. An important source of abietane diterpenes is *Rosmarinus officinalis* L. (rosemary), being carnosic acid and carnosol the main phenolic diterpenes from the leaves of this plant [5]. These compounds demonstrated antioxidant [6], antibacterial [7], antifungal [8,9], and cytotoxic activities [10]. A recent review of carnosol as an anticancer and antiinflammatory agent has been published [11].

In previous work, we investigated the gastroprotective activity and cytotoxicity of carnosic acid γ-lactone derivatives [12] as well as carnosic acid derivatives [13] and their gastroprotective mechanisms of action in human cells [14]. In the present report we used click chemistry reactions to prepare new carnosic acid and carnosol derivatives. Recent reports show that click chemistry is a very useful tool for drug discovery and gene therapy [15] that simplifies the synthesis of compounds through the use of simple and selective chemical transformations. Click chemistry reactions can be used for the generation of dimers, chimeras and multivalent drugs. The triazole in this case could be seen as an inactive linker or spacer, although it cannot be excluded that, at times, it may act as a biological entity on its own. 

Different compounds containing 1,2,3-triazoles with interesting antiproliferative activity have been reported [16,17,18,19,20]. This has recently led us to investigate the synthesis and antiproliferative activity of different terpenes coupled to triazole rings by the click chemistry technique [21,22,23]. The antifungal activity of triazoles is well known, being fluconazole, itraconazole, voriconazole and posaconazole the most used agents in the clinic [24]. However, their continued use has generated resistance from fungi making it necessary to find alternative antifungal compounds. Recent research has used click chemistry in the search for novel antifungal compounds [25,26,27,28]. Herein, we report an efficient method for the synthesis of novel carnosic acid and carnosol derivatives using click chemistry. The new compounds were assessed as antiproliferative and antifungal agents using human cell lines and reference microorganisms. 

## 2. Results and Discussion

**Scheme 1:**
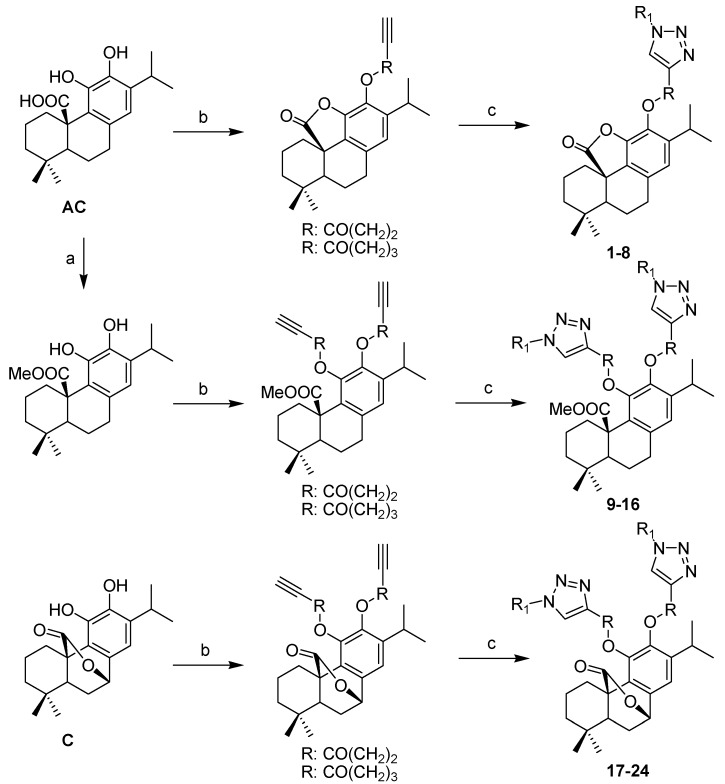
Preparation of carnosic acid and carnosol derivatives **1**–**24**.

A series of new abietane derivatives was synthesized by click chemistry. The diterpene carnosic acid (CA) was methylated using diazomethane in diethyl ether to obtain carnosic acid methyl ester (CAM). Previously we reported that treating CA with DCC/DMAP generated the corresponding carnosic acid γ-lactone (CAL) by an intramolecular esterification [12]. In this work six alkyl esters were prepared starting from CA, its methyl ester and carnosol (C), and then treated with different aromatic azides using click chemistry to produce 24 new compounds (Scheme 1).

**Table:** 

Compound	Compound	Compound	R	R_1_
1	9	17	CO(CH_2_)_2_	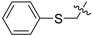
2	10	18	CO(CH_2_)_3_	
3	11	19	CO(CH_2_)_2_	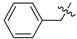
4	12	20	CO(CH_2_)_3_	
5	13	21	CO(CH_2_)_2_	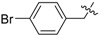
6	14	22	CO(CH_2_)_3_	
7	15	23	CO(CH_2_)_2_	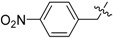
8	16	24	CO(CH_2_)_3_	

*Reagents and conditions*: (**a**) CH_2_N_2_, Et_2_O; (**b**) appropriate alkyne acid, DCC, DMAP CH_2_Cl_2_, 58%–76%; (**c**) appropriate azide, CuSO_4_·5H_2_O, sodium ascorbate, *t*-BuOH:H_2_O 1:1, 53%–83%.

Compounds **1**–**24** are described for the first time. All the products were characterized by spectroscopic means.

### 2.1. Antiproliferative Assay

The antiproliferative activity towards the following human cell lines was determined: normal lung fibroblasts (MRC-5), gastric epithelial adenocarcinoma (AGS), lung cancer (SK-MES-1) and bladder carcinoma (J82) cells. IC_50_ values > 100 µM were considered inactive. The hybrid compounds of carnosic acid γ-lactone (compound **1**–**8**) showed variable antiproliferative activity (Table 1). Compounds **1** and **2**, differing in the number of CH_2_ groups of the linker and presenting a methyl phenyl sulfide in the aromatic moiety showed about the same antiproliferative activity against MRC-5 (IC_50_ values 45.1 and 46.9 μg∙mL^−1^) and AGS cells (IC_50_ values 39.2 and 41.0 μg∙mL^−1^). Both compounds were also active against lung cancer cells SK-MES-1, with IC_50_ values of 81.7 and 76.0 μg∙mL^−1^, respectively. When comparing the pairs **3**–**4**, **5**–**6** and **7**–**8** differing in one CH_2_ unit in the linker, the activity decreased with linker length. The benzyl derivative **3** with two CH_2_ units in the linker was active towards MRC-5 and AGS cells, while the compound **4** presenting three CH_2_ units in the linker was inactive. 

For the carnosic acid methyl ester (compounds **9**–**16**) and carnosol (compounds **17**–**24**) derivatives, only compounds **11** and **23** showed weak antiproliferative activity against AGS cells (IC_50_ value: 89.4 μM and 99.4 µM respectively). All other compounds should be regarded as inactive on all cell lines tested. Overall, selectivity against MRC-5 and AGS cells was observed for some of the new compounds.

**Table 1:** Antiproliferative activity of carnosic acid γ-lactone derivatives **1**–**8** against MRC-5 normal fibroblasts and selected tumor cell lines *^a^*.

Compound	(IC_50_ ± SD, µM) ^*b*^			
	MRC-5	AGS	SK-MES-1	J82
**1**	45.1 ± 2.1	39.2 ± 2.3	81.7 ± 4.3	>100
**2**	46.9 ± 3.4	41.0 ± 2.1	76.0 ± 5.1	80.1 ± 4.3
**3**	43.4 ± 3.0	48.9 ± 3.9	73.0 ± 3.9	74.1 ± 3.9
**4**	>100	>100	>100	>100
**5**	82.6 ± 6.6	>100	>100	>100
**6**	>100	>100	>100	>100
**7**	60.6 ± 3.6	64.3 ± 4.5	>100	>100
**8**	>100	>100	>100	>100
Etoposide ^*c*^	0.33 ± 0.02	0.58 ± 0.02	1.83 ± 0.09	3.49 ± 0.16

^*a*^ Cell lines: normal lung fibroblasts (MRC-5), gastric epithelial adenocarcinoma (AGS), lung cancer (SK-MES-1) and bladder carcinoma (J82) cells; ^*b*^ Results are expressed as mean values ± SD. Each concentration was tested in sextuplicate together with the control and repeated two times in separate experiments; ^*c*^ Reference compound.

### 2.2. Antifungal Assays

The antifungal properties of compounds **1**–**24** against two clinical important fungal species, *C. albicans ATCC 10231 and C. neoformans ATCC 32264* were investigated. Results were expressed as the percentages of inhibition of each fungus in the range 250–3.9 μg∙mL^−1^ by using the standardized microbroth dilution method M-27A3 of Clinical and Laboratory Standards Institute [29] which assures reliable and reproducible results. Results are shown in Table 2 and Table 3.

The minimum inhibitory concentration of compound **1**–**24** necessary to completely inhibit (MIC_100_) the growth of the selected opportunistic pathogenic fungi was >250 μg∙mL^−1^. However, when considering less stringent end-points such as the minimum concentration required to inhibit 50% microbial growth (MIC_50_), there were interesting effects towards *C. albicans* ATCC 10231 and *C. neoformans* ATCC 32264.

From the results of Table 2 and Table 3, it is clear that *C. neoformans* is more sensitive than *C. albicans* to some members of the series. With regard to *C. albicans*, no compound displayed 80% inhibition at concentrations below 250 μg∙mL^−1^, being **2**, **4**, **12** and **18** moderately active (range 50.0%–57.9% inhibition) at 250 μg∙mL^−1^ whereas compounds **6**, **11** and **17** inhibited by 42.3%–45.7% of fungal growth at 250 μg∙mL^−1^.

On the other hand, compounds **2**, **4**, **9**–**12**, **14**, **22** and **23** inhibited >50% fungal growth (53.4%–91.3%) at 250 μg∙mL^−1^ against *C. neoformans* (Table 3) being **2**, **22** and **23** the most active ones with 71.6%–91.3% of fungal growth inhibition. Compounds **6**, **15**, **17**, **18**, **21** and **24** also showed interesting antifungal activities with 40.0%–45.8% growth inhibition. Compound **22** was the most active of the whole series against *C. neoformans*, with an inhibition of about 91.3% at 250 μg∙mL^−1^. 

From the results of Table 2, some structure/activity relationships can be inferred. Compounds **2**, **4**, **12** and **18**, that showed the best activities against *C. albicans*, possess the following common features (i) the linker to the diterpene moiety contained three CH_2_ units while the corresponding derivatives with two CH_2_ units were devoid of activity (compound **1** and **3**) or showed weak effect (compounds **11** and **17**) at the assayed concentrations; (ii) in the triazole rings, R_1_ was either a benzyl (compounds **4** and **12**) or a methyl phenyl sulfide (compounds **2** and **18**); (iii) the activity was almost the same for the four compounds, regardless of the presence of a lactone (carnosic acid γ-lactone derivatives **2** and **4** and carnosol derivative **18**) and one (carnosic acid γ-lactone derivatives **2** and **4**) or two triazole rings (carnosic acid methyl ester derivative **12** and carnosol derivative **18**); (iv) when R_1_ joined to the triazole ring was *p*-bromobenzyl or *p*-nitrobenzyl, the corresponding derivatives were inactive.

The structure/activity trends observed for the compounds on *C. neoformans* indicate that the nature of the substituent on the triazole ring is relevant for the effect and different than for *C. albicans*. For compound **2** (74.8% inhibition growth at 250 μg∙mL^−1^) and **4** (53.4%) the γ-lactone appears to be important for activity. In the carnosic acid methyl ester derivatives group, compounds **9**–**12** and **14** were active in the range 57.2%–67.4% inhibition at 250 μg∙mL^−1^. For the pairs **9**–**10** (R_1_: methyl phenyl sulfide) and **11**–**12** (R_1_: benzyl) bearing two or three CH_2_ units as linkers, the effect was similar.

When comparing **11**–**12** with **13**–**14** (R_1_: *p*-bromobenzyl), the occurrence of a bromine in the aromatic ring did not change the activity when the length of the linker is three CH_2_ units, but it diminishes when the linker contains two CH_2_ units. When comparing the activity of **11**–**12** with **15**–**16**, presenting a nitro group in the aromatic ring (R_1_: *p*-nitrobenzyl), the activity of the nitro compounds is lower.

**Table 2:** Inhibition percentages displayed by **1**–**24** against *C. albicans* ATCC 10231 at the concentrations range 250–3.9 μg∙mL^−1^. The minimum concentrations of all compounds necessary to inhibit 50% of fungal growth (MIC_50_) were included in the table. Standard drug: amphotericin B (Amph B).

Compound	250 μg∙mL^−1^	125 μg∙mL^−1^	62.5 μg∙mL^−1^	31. μg∙mL^−1^	15.6 μg∙mL^−1^	7.8 μg∙mL^−1^	3.9 μg∙mL^−1^	MIC_50_ in μg∙mL^−1^
**1**	17.1 ± 0.1	8.2 ± 0.1	3.5 ± 0.3	1.9 ± 0.3	0.4 ± 0.2	0.0	0.0	>250
**2**	50.7 ± 0.3	27.9 ± 1.8	15.7 ± 1.8	8.9 ± 1.8	5.2 ± 0.6	1.9 ± 0.5	1.5 ± 0.1	250
**3**	20.3 ± 3.9	20.2 ± 2.2	14.3 ± 2.0	11.1 ± 0.4	9.1 ± 1.5	8.1 ± 0.8	5.7 ± 0.8	>250
**4**	51.6 ± 0.1	28.2 ± 2.1	16.4 ± 2.3	10.2 ± 1.2	9.5 ± 1.3	4.1 ± 1.7	3.7 ± 0.4	250
**5**	16.5 ± 1.3	8.3 ± 3.3	4.1 ± 1.7	3.0 ± 2.7	0.0	0.0	0.0	>250
**6**	45.4 ± 3.4	19.9 ± 0.5	13.2 ± 1.4	4.2 ± 0.9	1.5 ± 0.1	1.4 ± 0.4	0.0	>250
**7**	15.5 ± 1.1	10.5 ± 3.0	2.5 ± 0.5	2.2 ± 0.7	2.1 ± 0.1	0.9 ± 0.2	0.0	>250
**8**	27.5 ± 2.0	14.0 ± 1.9	5.6 ± 1.7	2.5 ± 1.3	0.0	0.0	0.0	>250
**9**	15.9 ± 1.2	19.2 ± 0.1	9.4 ± 0.7	4.7 ± 0.1	3.5 ± 0.3	0.0	0.0	>250
**10**	35.0 ± 0.8	18.8 ± 0.4	10.1 ± 0.9	5.1 ± 1.3	3.9 ± 0.9	2.8 ± 0.1	1.3 ± 1.3	>250
**11**	45.7 ± 3.0	23.3 ± 2.1	11.8 ± 0.3	5.5 ± 1.7	3.5 ± 0.7	1.9 ± 0.5	1.8 ± 0.1	>250
**12**	50.0 ± 5.0	27.9 ± 0.1	14.9 ± 1.1	9.3 ± 2.6	5.6 ± 1.9	3.6 ± 0.4	3.4 ± 0.3	250
**13**	17.6 ± 0.2	10.7 ± 0.3	6.7 ± 0.1	4.3 ± 0.4	1.3 ± 0.3	0.6 ± 0.1	0.0	>250
**14**	29.1 ± 2.5	13.3 ± 0.6	9.3 ± 1.5	6.3 ± 1.5	1.9 ± 0.3	1.3 ± 0.3	1.3 ± 0.2	>250
**15**	33.4 ± 2.7	15.2 ± 2.8	8.2 ± 0.3	3.0 ± 1.1	2.6 ± 0.3	0.0	0.0	>250
**16**	21.1 ± 2.4	11.7 ± 0.5	7.2 ± 0.1	3.4 ± 0.5	2.3 ± 0.1	0.8 ± 0.1	0.0	>250
**17**	42.3 ± 0.7	21.2 ± 0.2	11.5 ± 0.7	6.7 ± 1.1	4.8 ± 1.0	2.8 ± 0.6	0.0	>250
**18**	57.9 ± 1.0	35.4 ± 2.1	18.3 ± 0.1	11.5 ± 1.8	2.8 ± 1.0	2.5 ± 0.2	1.0 ± 0.1	250
**19**	37.8 ± 0.5	21.1 ± 1.0	11.9 ± 1.0	4.6 ± 1.8	3.2 ± 0.1	3.2 ± 0.1	0.7 ± 0.3	>250
**20**	34.5 ± 0.5	21.4 ± 3.4	10.9 ± 1.6	4.3 ± 0.4	3.0 ± 0.4	1.6 ± 0.2	0.7 ± 0.1	>250
**21**	8.6 ± 2.0	6.6 ± 1.1	4.4 ± 1.3	3.5 ± 0.6	2.6 ± 0.6	0.0	0.0	>250
**22**	12.5 ± 2.2	10.1 ± 0.8	7.0 ± 1.0	5.9 ± 0.2	5.1 ± 0.4	2.9 ± 0.7	0.0	>250
**23**	25.5 ± 2.5	17.6 ± 1.4	6.7 ± 1.6	6.0 ± 0.5	3.3 ± 0.6	1.6 ± 0.8	0.0	>250
**24**	29.8 ± 1.9	14.3 ± 2.0	4.8 ± 0.6	3.4 ± 0.5	0.0	0.0	0.0	>250
Amph B	100	100	100	100	100	100	100	0.25

**Table 3:** Inhibition percentages displayed by **1**–**24** against *C. neoformans* ATCC 32264 at the concentrations range 250–3.9 μg∙mL^−1^. The minimum concentrations of all compounds necessary to inhibit 50% of fungal growth (MIC_50_) were included in the table. Standard drug: amphotericin B (Amph B).

Compound	250 μg∙mL^−1^	125 μg∙mL^−1^	62.5 μg∙mL^−1^	31.2 μg∙mL^−1^	15.6 μg∙mL^−1^	7.8 μg∙mL^−1^	3.9 μg∙mL^−1^	MIC_50_ in μg∙mL^−1^
**1**	11.0 ± 0.5	8.9 ± 0.7	3.9 ± 0.4	3.7 ± 0.1	0.0	0.0	0.0	>250
**2**	74.8 ± 2.8	22.1 ± 1.5	20.7 ± 0.6	11.8 ± 0.3	10.3 ± 1.0	1.9 ± 0.5	1.5 ± 0.1	250
**3**	26.7 ± 1.5	18.4 ± 0.2	16.7 ± 0.5	14.3 ± 2.0	9.7 ± 0.1	9.0 ± 0.3	4.9 ± 0.5	>250
**4**	53.4 ± 2.1	17.8 ± 1.4	9.8 ± 1.1	8.5 ± 1.0	2.3 ± 1.0	0.0	0.0	250
**5**	36.9 ± 2.2	12.6 ± 0.5	12.0 ± 1.0	8.7 ± 1.4	7.7 ± 0.6	6.0 ± 0.7	2.3 ± 0.9	>250
**6**	41.4 ± 2.7	22.4 ± 1.5	12.7 ± 0.8	5.4 ± 0.7	0.0	0.0	0.0	>250
**7**	28.2 ± 2.7	20.0 ± 1.1	0.0	0.0	0.0	0.0	0.0	>250
**8**	10.4 ± 2.8	9.5 ± 2.9	8.2 ± 2.8	0.8 ± 0.1	0.3 ± 0.1	0.0	0.0	>250
**9**	62.4 ± 3.8	53.6 ± 2.6	23.4 ± 1.3	13.7 ± 1.5	7.3 ± 0.4	5.1 ± 0.7	0.0	125
**10**	63.9 ± 2.9	42.9 ± 2.0	19.1 ± 1.8	18.6 ± 1.5	14.4 ± 0.8	11.3 ± 0.5	0.0	250
**11**	57.2 ± 2.4	38.3 ± 2.9	19.4 ± 2.1	14.2 ± 1.3	13.3 ± 2.0	2.7 ± 0.9	0.0	250
**12**	63.1 ± 2.1	67.5 ± 0.7	17.3 ± 0.2	9.6 ± 0.8	7.7 ± 1.6	0.0	0.0	125
**13**	25.7 ± 0.5	16.8 ± 1.8	16.3 ± 1.1	13.7 ± 0.6	13.3 ± 0.3	10.0 ± 1.0	5.8 ± 0.6	>250
**14**	67.4 ± 1.7	30.1 ± 1.4	16.9 ± 1.1	12.0 ± 1.2	10.1 ± 1.0	4.5 ± 0.2	0.0	250
**15**	45.4 ± 3.0	22.0 ± 2.7	15.5 ± 2.0	13.0 ± 1.4	10.0 ± 0.6	0.0	0.0	>250
**16**	36.9 ± 0.8	35.5 ± 0.4	14.5 ± 0.2	7.9 ± 0.1	5.0 ± 0.4	3.5 ± 0.4	0.0	>250
**17**	41.1 ± 1.3	11.4 ± 1.2	0.0	0.0	0.0	0.0	0.0	>250
**18**	44.3 ± 1.9	11.8 ± 0.6	0.0	0.0	0.0	0.0	0.0	>250
**19**	31.3 ± 1.6	7.1 ± 2.0	3.3 ± 1.1	0.0	0.0	0.0	0.0	>250
**20**	22.0 ± 1.0	14.5 ± 1.2	10.4 ± 0.2	2.7 ± 2.7	0.0	0.0	0.0	>250
**21**	40.0 ± 0.3	28.8 ± 0.4	19.2 ± 0.2	18.9 ± 0.7	17.9 ± 0.2	6.7 ± 0.3	1.2 ± 0.6	>250
**22**	91.3 ± 3.0	52.3 ± 2.3	28.5 ± 1.3	7.6 ± 0.9	4.8 ± 0.2	4.7 ± 0.8	0.0	125
**23**	71.6 ± 2.2	37.7 ± 0.7	32.6 ± 0.8	18.1 ± 0.4	16.2 ± 0.2	11.5 ± 1.5	6.0 ± 0.1	250
**24**	45.8 ± 2.0	31.7 ± 1.6	27.3 ± 0.7	24.3 ± 0.5	21.0 ± 0.1	15.4 ± 0.1	4.9 ± 1.4	>250
Amph B	100	100	100	100	100	100	100	0.50

The most active carnosol derivative was the *p*-bromobenzyl derivative **22**, which reduced the growth of *C. neoformans* by about 91% at 250 μg∙mL^−1^ while compound **23**, with a *p*-nitrobenzyl unit decreased fungal growth by about 71% at the same concentration. The results indicate some selectivity for the different fungi and that the placement of the lactone (either C-20, C-11 or C-20, C-7) is important for the effect. Further studies including additional biological models are advisable to find novel activities for the new synthetic compounds.

## 3. Experimental Section

### 3.1. General Procedures

Melting points were determined on a Koffler hot stage apparatus (Electrothermal 9100, Dubuque, IA, USA) and were uncorrected. Optical rotations were measured on a Jasco DIP 370 (Jasco Analytical Instruments, Easton, MD, USA) polarimeter in CHCl_3_ at 20 °C. IR spectra were recorded on a Nicolet Nexus 470 FT-IR instrument (Thermo Electron Corporation, Waltham, MA, USA). The NMR spectra were recorded in CDCl_3_ on a Bruker Avance 400 (Bruker, Rheinstetten, Germany) spectrometer at 400 MHz for ^1^H and 100 MHz for ^13^C. Chemical shifts are given in ppm with TMS as the internal standard. High-resolution mass spectra were measured on a VG Micromass ZAB-2F at 70 eV (Varian Inc., Palo Alto, CA, USA). Merck silica gel (0.063–0.2) was used for column chromatography, pre-coated Si gel plates (Merck, Kieselgel 60 F_254_, 0.25 mm) were used for TLC analysis. TLC spots were visualized by spraying the chromatograms with *p*-anisaldehyde–ethanol–acetic acid-H_2_SO_4_ (2:170:20:10 *v*/*v*) and heating at 110 °C for 3 min. Reagents: *N*,*N*-Dicyclohexylcarbodiimide (DCC) and dimethylaminopyridine (DMAP) were from Merck (Schuchardt, Germany). 4-Pentynoic acid, 5-hexynoic acid and aromatic azides were from Aldrich (Schuchardt, Germany). Copper (II) sulphate pentahydrate was from Aldrich (St. Louis, MO, USA) and sodium ascorbate was from Sigma (St. Louis, MO, USA).

### 3.2. General Procedure for the Synthesis of Compounds **1**–**24**

Carnosol and carnosic acid (CA) were isolated from the aerial parts of *Rosmarinus officinalis* as described previously [12]. Methylation of CA was performed using diazomethane in diethyl ether (Et_2_O). The compounds **1**–**24** were prepared treating carnosol, carnosic acid and carnosic acid methyl ester with the appropriate alkyne acid/DCC/DMAP to obtain the esters. Treatment with the appropriate azide yielded the corresponding triazole.

#### 3.2.1. Preparation of Alkynyl Esters

Esterification of carnosol, carnosic acid and carnosic acid methyl ester was performed using DCC/DMAP and appropriate acid (4-pentynoic acid or 5-hexynoic acid) according to references [22,23]. Briefly, alkynyl acid (1 eq) was dissolved in dry CH_2_Cl_2_ at room temperature under constant stirring. Then, DCC (1 eq) was added, followed by a catalytic amount of DMAP and the corresponding terpene (0.5 eq) dissolved in dry CH_2_Cl_2_. The reaction was stopped by adding H_2_O, extracted with CH_2_Cl_2_, dried over Na_2_SO_4_, concentrated and purified (58%–76% yield).

#### 3.2.2. General Procedure for the Synthesis of Triazoles

The alkynyl esters (1 eq) and the corresponding azide (1 eq) were dissolved in *t*-BuOH/H_2_O (1:1), followed by the addition of CuSO_4_·5H_2_O (2 mol %) and sodium ascorbate (10 mol %). The mixture was stirred at room temperature for 24 h. The reaction was stopped by adding H_2_O, extracted with CH_2_Cl_2_, dried over anhydrous Na_2_SO_4_, concentrated and purified by column chromatography on silica gel (53%–83% yield).

*12-O-(3-(((1-phenylthio)methyl)-1H-1,2,3-triazol-4-yl)-propanoyloxy)-11,20-epoxyabieta-8,11,13-trien-20-one* (**1**). Pale yellow resin; ${\left[\alpha \right]}_{D}^{20}$ +16 (*c* 0.227, CHCl_3_); IR ν_max_ (film) 3142, 2950, 2864, 1798, 1754, 1439, 1130, 755 cm^−1^; ^1^H-NMR (CDCl_3_): δ 7.56 (1H, s, H-5′), 7.31–7.33 (2H, m, H-2′′ and H-6′′), 7.25–7.28 (3H, m, H-3′′; H-4′′ and H-5′′), 6.72 (1H, s, H-14), 5.63 (2H, s, CH_2_S), 3.16 (2H, t, *J =* 6.9 Hz, H-3′), 2.99 (2H, t, *J =* 6.9 Hz, H-2′), 2.97 (1H, m, H-15), 2.61 (2H, m, H-7), 2.24 (1H, m, H-1), 2.09 (1H, m, H-3), 2.00 (1H, m, H-2), 1.88–1.95 (2H, m, H-5 and H-6), 1.83 (1H, m, H-1), 1.70 (1H, m, H-2), 1.39 (1H, m, H-3), 1.19 (3H, d, *J =* 6.9 Hz, H-16), 1.16 (3H, s, H-18), 1.12 (3H, d, *J =* 6.9 Hz, H-17), 1.08 (3H, s, H-19), 0.87 (1H, m, H-6); ^13^C-NMR (CDCl_3_): δ 42.0 (C-1), 18.6 (C-2), 39.1 (C-3), 33.2 (C-4), 56.9 (C-5), 24.3 (C-6), 33.3 (C-7), 137.7 (C-8), 130.8 (C-9), 50.0 (C-10), 144.9 (C-11), 128.9 (C-12), 141.2 (C-13), 121.9 (C-14), 28.1 (C-15), 23.0 (C-16), 23.9 (C-17), 32.2 (C-18), 22.6 (C-19), 178.0 (C-20), 170.5 (C-1′), 33.9 (C-2′), 21.6 (C-3′), 146.8 (C-4′), 120.4 (C-5′), 54.1 (CH_2_S), 132.6 (C-1′′), 132.7 (2C, C-2′′ and C-6′′), 129.8 (2C, C-3′′ and C-5′′), 129.8 (C-4′′); EIMS *m/z* 532.2560 [M+H-CO]^+^ (calcd for C_31_H_38_N_3_O_3_S, 532.2634).

*12-O-(4-(((1-phenylthio)methyl)-1H-1,2,3-triazol-4-yl)-butanoyloxy)-11,20-epoxyabieta-8,11,13-trien-20-one* (**2**). Pale yellow resin; ${\left[\alpha \right]}_{D}^{20}$ +28 (*c* 0.124, CHCl_3_); IR ν_max_ (film) 3143, 2952, 2870, 1798, 1757, 1437, 1126, 750 cm^−1^; ^1^H-NMR (CDCl_3_): δ 7.41 (1H, s, H-6′), 7.31–7.33 (2H, m, H-2′′ and H-6′′), 7.25–7.28 (3H, m, H-3′′; H-4′′ and H-5′′), 6.72 (1H, s, H-14), 5.59 (2H, s, CH_2_S), 3.00 (1H, m, H-15), 2.83 (2H, t, *J =* 7.4 Hz, H-4′), 2.59–2.61 (4H, m, H-7 and H-2′), 2.24 (1H, m, H-1), 2.05–2.14 (3H, m, H-3 and H-3′), 2.00 (1H, m, H-2), 1.87–1.95 (2H, m, H-5 and H-6), 1.82 (1H, m, H-1), 1.68 (1H, m, H-2), 1.37 (1H, m, H-3), 1.20 (3H, d, *J =* 6.9 Hz, H-16), 1.15 (3H, d, *J =* 6.9 Hz, H-17), 1.14 (3H, s, H-18), 1.07 (3H, s, H-19), 0.86 (1H, m, H-6); ^13^C-NMR (CDCl_3_): δ 41.4 (C-1), 18.1 (C-2), 38.5 (C-3), 32.7 (C-4), 56.3 (C-5), 24.3 (C-6), 32.7 (C-7), 137.1 (C-8), 130.3 (C-9), 49.5 (C-10), 144.5 (C-11), 128.6 (C-12), 140.7 (C-13), 120.9 (C-14), 27.6 (C-15), 23.4 (C-16), 23.8 (C-17), 31.7 (C-18), 22.5 (C-19), 177.5 (C-20), 170.5 (C-1′), 32.7 (C-2′), 22.1 (C-3′), 24.4 (C-4′), 147.2 (C-5′), 119.8 (C-6′), 53.6 (CH_2_S), 129.5 (C-1′′), 132.3 (2C, C-2′′ and C-6′′), 129.3 (2C, C-3′′ and C-5′′), 131.8 (C-4′′); EIMS *m/z* 546.2603 [M+H]^+^ (calcd for C_32_H_40_N_3_O_3_S, 546.2790).

*12-O-(3-(1-benzyl-1H-1,2,3-triazol-4-yl)-propanoyloxy)-11,20-epoxyabieta-8,11,13-trien-20-one* (**3**). White resin; mp 154 °C; ${\left[\alpha \right]}_{D}^{20}$ +40 (*c* 0.096, CHCl_3_); IR ν_max_ (film) 3150, 2961, 2870, 1795, 1760, 1431, 1130, 753 cm^−1^; ^1^H-NMR (CDCl_3_): δ 7.51 (1H, s, H-5′), 7.32–7.34 (3H, m, H-2′′; H-4′′ and H-6′′), 7.25–7.28 (2H, m, H-3′′ and H-5′′), 6.74 (1H, s, H-14), 5.53 (2H, s, CH_2_Ph), 3.18 (2H, t, *J =* 6.9 Hz, H-3′), 3.00 (2H, t, *J =* 6.9 Hz, H-2′), 2.98 (1H, m, H-15), 2.63 (2H, m, H-7), 2.24 (1H, m, H-1), 2.10 (1H, m, H-3), 2.01 (1H, m, H-2), 1.88–1.96 (2H, m, H-5 and H-6), 1.84 (1H, m, H-1), 1.72 (1H, m, H-2), 1.41 (1H, m, H-3), 1.20 (3H, d, *J =* 6.9 Hz, H-16), 1.18 (3H, s, H-18), 1.13 (3H, d, *J =* 6.9 Hz, H-17), 1.10 (3H, s, H-19), 0.88 (1H, m, H-6); ^13^C-NMR (CDCl_3_): δ 42.0 (C-1), 18.6 (C-2), 39.1 (C-3), 33.2 (C-4), 56.9 (C-5), 24.3 (C-6), 33.3 (C-7), 137.7 (C-8), 130.8 (C-9), 50.0 (C-10), 144.9 (C-11), 129.8 (C-12), 141.3 (C-13), 122.2 (C-14), 28.1 (C-15), 23.0 (C-16), 23.9 (C-17), 32.2 (C-18), 22.6 (C-19), 178.0 (C-20), 170.6 (C-1′), 33.9 (C-2′), 21.5 (C-3′), 146.7 (C-4′), 120.4 (C-5′), 54.4 (CH_2_Ph), 135.5 (C-1′′), 129.4 (2C, C-2′′ and C-6′′), 128.4 (2C, C-3′′ and C-5′′), 128.9 (C-4′′); EIMS *m/z* 500.2890 [M+H]^+^ (calcd for C_31_H_38_N_3_O_3_, 500.2913).

*12-O-(4-(1-benzyl-1H-1,2,3-triazol-4-yl)-butanoyloxy)-11,20-epoxyabieta-8,11,13-trien-20-one* (**4**). White resin; mp 162 °C; ${\left[\alpha \right]}_{D}^{20}$ +51 (*c* 0.106, CHCl_3_); IR ν_max_ (film) 3141, 2950, 2868, 1793, 1754, 1440, 1128, 753 cm^−1^; ^1^H-NMR (CDCl_3_): δ 7.43 (1H, s, H-6′), 7.33–7.35 (3H, m, H-2′′; H-4′′ and H-6′′), 7.26–7.29 (2H, m, H-3′′ and H-5′′), 6.73 (1H, s, H-14), 5.49 (2H, s, CH_2_Ph), 3.01 (1H, m, H-15), 2.84 (2H, t, *J =* 7.4 Hz, H-4′), 2.59–2.62 (4H, m, H-7 and H-2′), 2.25 (1H, m, H-1), 2.06–2.15 (3H, m, H-3 and H-3′), 2.00 (1H, m, H-2), 1.88–1.96 (2H, m, H-5 and H-6), 1.83 (1H, m, H-1), 1.68 (1H, m, H-2), 1.38 (1H, m, H-3), 1.21 (3H, d, *J =* 6.9 Hz, H-16), 1.15 (3H, d, *J =* 6.9 Hz, H-17), 1.14 (3H, s, H-18), 1.08 (3H, s, H-19), 0.86 (1H, m, H-6); ^13^C-NMR (CDCl_3_): δ 41.5 (C-1), 18.1 (C-2), 38.7 (C-3), 33.1 (C-4), 56.8 (C-5), 24.3 (C-6), 32.3 (C-7), 137.5 (C-8), 130.7 (C-9), 49.9 (C-10), 144.5 (C-11), 129.4 (C-12), 140.9 (C-13), 121.8 (C-14), 27.6 (C-15), 23.0 (C-16), 23.9 (C-17), 32.2 (C-18), 22.6 (C-19), 177.6 (C-20), 170.5 (C-1′), 32.7 (C-2′), 22.1 (C-3′), 24.3 (C-4′), 147.5 (C-5′), 119.6 (C-6′), 53.5 (CH_2_Ph), 135.3 (C-1′′), 129.5 (2C, C-2′′ and C-6′′), 128.4 (2C, C-3′′ and C-5′′), 129.1 (C-4′′); EIMS *m/z* 514.3124 [M+H]^+^ (calcd for C_32_H_40_N_3_O_3_, 514.3070).

*12-O-(3-(1-(4-bromobenzyl)-1H-1,2,3-triazol-4-yl)-propanoyloxy)-11,20-epoxyabieta-8,11,13-trien-20-one* (**5**). White resin; ${\left[\alpha \right]}_{D}^{20}$ +61 (*c* 0.131, CHCl_3_); IR ν_max_ (film) 3150, 2961, 2867, 1798, 1762, 1442, 1129, 763 cm^−1^; ^1^H-NMR (CDCl_3_): δ 7.54 (1H, s, H-5′), 7.41 (2H, d, *J =* 8.4 Hz, H-3′′ and H-5′′), 7.12 (2H, d, *J =* 8.4 Hz, H-2′′ and H-6′′), 6.71 (1H, s, H-14), 5.47 (2H, s, CH_2_PhBr), 3.17 (2H, t, *J =* 6.8 Hz, H-3′), 2.98 (2H, t, *J =* 6.8 Hz, H-2′), 2.96 (1H, m, H-15), 2.60 (2H, m, H-7), 2.18 (1H, m, H-1), 2.06 (1H, m, H-3), 1.97 (1H, m, H-2), 1.86–1.94 (2H, m, H-5 and H-6), 1.81 (1H, m, H-1), 1.70 (1H, m, H-2), 1.38 (1H, m, H-3), 1.17 (3H, d, *J =* 6.9 Hz, H-16), 1.14 (3H, s, H-18), 1.10 (3H, d, *J =* 6.9 Hz, H-17), 1.07 (3H, s, H-19), 0.86 (1H, m, H-6); ^13^C-NMR (CDCl_3_): δ 42.0 (C-1), 18.6 (C-2), 39.0 (C-3), 33.2 (C-4), 56.8 (C-5), 24.3 (C-6), 33.3 (C-7), 137.7 (C-8), 130.7 (C-9), 50.0 (C-10), 144.8 (C-11), 129.7 (C-12), 141.2 (C-13), 122.3 (C-14), 28.1 (C-15), 23.0 (C-16), 23.9 (C-17), 32.2 (C-18), 22.6 (C-19), 178.1 (C-20), 170.6 (C-1′), 34.0 (C-2′), 21.6 (C-3′), 146.8 (C-4′), 120.5 (C-5′), 53.6 (CH_2_PhBr), 134.7 (C-1′′), 130.0 (2C, C-2′′ and C-6′′), 132.5 (2C, C-3′′ and C-5′′), 123.0 (C-4′′); EIMS *m/z* 578.2042 [M+H]^+^ (calcd for C_31_H_37_BrN_3_O_3_, 578.2018).

*12-O-(4-(1-(4-bromobenzyl)-1H-1,2,3-triazol-4-yl)-butanoyloxy)-11,20-epoxyabieta-8,11,13-trien-20-one* (**6**). White resin; pale yellow resin; ${\left[\alpha \right]}_{D}^{20}$ +54 (*c* 0.088, CHCl_3_); IR ν_max_ (film) 3144, 2943, 2862, 1796, 1766, 1446, 1126, 753 cm^−1^; ^1^H-NMR (CDCl_3_): δ 7.42 (1H, s, H-6′), 7.40 (2H, d, *J =* 8.4 Hz, H-3′′ and H-5′′), 7.10 (2H, d, *J =* 8.4 Hz, H-2′′ and H-6′′), 6.70 (1H, s, H-14), 5.44 (2H, s, CH_2_PhBr), 3.00 (1H, m, H-15), 2.86 (2H, t, *J =* 7.4 Hz, H-4′), 2.59–2.62 (4H, m, H-7 and H-2′), 2.26 (1H, m, H-1), 2.07–2.15 (3H, m, H-3 and H-3′), 2.03 (1H, m, H-2), 1.88–1.96 (2H, m, H-5 and H-6), 1.83 (1H, m, H-1), 1.69 (1H, m, H-2), 1.38 (1H, m, H-3), 1.20 (3H, d, *J =* 6.9 Hz, H-16), 1.16 (3H, d, *J =* 6.9 Hz, H-17), 1.15 (3H, s, H-18), 1.10 (3H, s, H-19), 0.87 (1H, m, H-6); ^13^C-NMR (CDCl_3_): δ 41.7 (C-1), 18.5 (C-2), 38.8 (C-3), 32.1 (C-4), 56.4 (C-5), 24.5 (C-6), 32.3 (C-7), 137.7 (C-8), 130.7 (C-9), 50.0 (C-10), 144.7 (C-11), 129.6 (C-12), 140.9 (C-13), 121.9 (C-14), 27.9 (C-15), 23.1 (C-16), 23.8 (C-17), 32.1 (C-18), 22.6 (C-19), 177.6 (C-20), 170.4 (C-1′), 32.8 (C-2′), 22.2 (C-3′), 24.4 (C-4′), 147.3 (C-5′), 119.8 (C-6′), 53.4 (CH_2_PhBr), 134.3 (C-1′′), 130.1 (2C, C-2′′ and C-6′′), 132.7 (2C, C-3′′ and C-5′′), 122.6 (C-4′′); EIMS *m/z* 592.2296 [M+H]^+^ (calcd for C_32_H_39_BrN_3_O_3_, 592.2175).

*12-O-(3-(1-(4-nitrobenzyl)-1H-1,2,3-triazol-4-yl)-propanoyloxy)-11,20-epoxyabieta-8,11,13-trien-20-one* (**7**). Colorless resin; ${\left[\alpha \right]}_{D}^{20}$ +24 (*c* 0.107, CHCl_3_); IR ν_max_ (film) 3139, 2954, 2870, 1791, 1758, 1436, 1124, 752 cm^−1^; ^1^H-NMR (CDCl_3_): δ 8.13 (2H, d, *J =* 8.7 Hz, H-3′′ and H-5′′), 7.68 (1H, s, H-5′), 7.38 (2H, d, *J =* 8.7 Hz, H-2′′ and H-6′′), 6.72 (1H, s, H-14), 5.66 (2H, s, CH_2_PhNO_2_), 3.20 (2H, t, *J =* 6.8 Hz, H-3′), 2.98 (2H, t, *J =* 6.8 Hz, H-2′), 2.96 (1H, m, H-15), 2.60 (2H, m, H-7), 2.16 (1H, m, H-1), 2.04 (1H, m, H-3), 1.96 (1H, m, H-2), 1.85–1.94 (2H, m, H-5 and H-6), 1.81 (1H, m, H-1), 1.69 (1H, m, H-2), 1.38 (1H, m, H-3), 1.17 (3H, d, *J =* 6.9 Hz, H-16), 1.12 (3H, s, H-18), 1.10 (3H, d, *J =* 6.9 Hz, H-17), 1.06 (3H, s, H-19), 0.84 (1H, m, H-6); ^13^C-NMR (CDCl_3_): δ 42.0 (C-1), 18.5 (C-2), 39.0 (C-3), 33.1 (C-4), 56.8 (C-5), 24.3 (C-6), 33.2 (C-7), 137.8 (C-8), 130.7 (C-9), 50.0 (C-10), 144.7 (C-11), 129.7 (C-12), 141.2 (C-13), 122.8 (C-14), 28.1 (C-15), 22.9 (C-16), 23.8 (C-17), 32.2 (C-18), 22.5 (C-19), 78.2 (C-20), 170.5 (C-1′), 34.0 (C-2′), 21.7 (C-3′), 147.1 (C-4′), 120.6 (C-5′), 53.2 (CH_2_PhNO_2_), 142.8 (C-1′′), 129.0 (2C, C-2′′ and C-6′′), 124.4 (2C, C-3′′ and C-5′′), 148.3 (C-4′′); EIMS *m/z* 545.2931 [M+H]^+^ (calcd for C_31_H_37_N_4_O_5_, 545.2764). 

*12-O-(4-(1-(4-nitrobenzyl)-1H-1,2,3-triazol-4-yl)-butanoyloxy)-11,20-epoxyabieta-8,11,13-trien-20-one* (**8**). Colorless resin; ${\left[\alpha \right]}_{D}^{20}$ +30 (*c* 0.072, CHCl_3_); IR ν_max_ (film) 3133, 2950, 2864, 1795, 1761, 1442, 1130, 755 cm^−1^; ^1^H-NMR (CDCl_3_): δ 8.15 (2H, d, *J =* 8.7 Hz, H-3′′ and H-5′′), 7.56 (1H, s, H-6′), 7.39 (2H, d, *J =* 8.7 Hz, H-2′′ and H-6′′), 6.70 (1H, s, H-14), 5.63 (2H, s, CH_2_PhNO_2_), 2.98 (1H, m, H-15), 2.84 (2H, t, *J =* 7.4 Hz, H-4′), 2.57–2.60 (4H, m, H-7 and H-2′), 2.24 (1H, m, H-1), 2.06–2.14 (3H, m, H-3 and H-3′), 2.00 (1H, m, H-2), 1.87–1.94 (2H, m, H-5 and H-6), 1.82 (1H, m, H-1), 1.69 (1H, m, H-2), 1.37 (1H, m, H-3), 1.19 (3H, d, *J =* 6.9 Hz, H-16), 1.15 (3H, d, *J =* 6.9 Hz, H-17), 1.13 (3H, s, H-18), 1.06 (3H, s, H-19), 0.84 (1H, m, H-6); ^13^C-NMR (CDCl_3_): δ 41.3 (C-1), 18.6 (C-2), 38.8 (C-3), 32.4 (C-4), 56.6 (C-5), 24.3 (C-6), 32.5 (C-7), 137.1 (C-8), 130.7 (C-9), 49.9 (C-10), 144.6 (C-11), 128.9 (C-12), 140.8 (C-13), 121.7 (C-14), 27.7 (C-15), 23.4 (C-16), 23.8 (C-17), 32.2 (C-18), 22.5 (C-19), 177.5 (C-20), 170.6 (C-1′), 32.7 (C-2′), 22.3 (C-3′), 24.3 (C-4′), 147.1 (C-5′), 119.4 (C-6′), 53.4 (CH_2_ PhNO_2_), 142.4 (C-1′′), 129.0 (2C, C-2′′ and C-6′′), 129.6 (2C, C-3′′ and C-5′′), 148.0 (C-4′′); EIMS *m/z* 559.3044 [M+H]^+^ (calcd for C_32_H_39_N_4_O_5_, 559.2920). 

*Methyl(11,12-O-(3-(((1-phenylthio)methyl)-1H-1,2,3-triazol-4-yl)-propanoyloxy)-abieta-8,11,13-triene)-20-oate* (**9**). Pale yellow resin; ${\left[\alpha \right]}_{D}^{20}$ +18 (*c* 0.053, CHCl_3_); IR ν_max_ (film) 3147, 2967, 2873, 1769, 1743, 1458, 1112, 760 cm^−1^; ^1^H-NMR (CDCl_3_): δ 7.51, 7.49 (each 1H, s, H-5′), 7.26–7.30 (10H, m, 2×SPh), 6.92 (1H, s, H-14), 5.54 (4H, brs, 2×CH_2_S), 3.45 (3H, s, COOMe), 3.22 (1H, brd, *J =* 13.0 Hz, H-1), 2.97–3.04 (4H, m, 2×H-3′), 2.90–2.95 (2H, m, H-7), 2.73–2.88 (5H, m, H-15 and 2×H-2′), 2.28 (1H, m, H-6), 2.06 (1H, m, H-2), 1.83 (1H, m, H-6), 1.43–1.52 (3H, m, H-2; H-3 and H-5), 1.19–1.27 (2H, m, H-1 and H-3), 1.13 (3H, d, *J =* 6.9 Hz, H-16), 1.03 (3H, d, *J =* 6.9 Hz, H-17), 0.95 (3H, s, H-18), 0.72 (3H, s, H-19); ^13^C-NMR (CDCl_3_): δ 34.6 (C-1), 20.4 (C-2), 40.9 (C-3), 33.8 (C-4), 53.4 (C-5), 19.6 (C-6), 31.7 (C-7), 136.6 (C-8), 131.5 (C-9), 47.5 (C-10), 141.1 (C-11), 138.3 (C-12), 139.6 (C-13), 124.9 (C-14), 27.1 (C-15), 22.4 (C-16), 22.9 (C-17), 32.3 (C-18), 19.7 (C-19), 175.1 (C-20), 51.6 (OMe), 170.2, 169.9 (C-1′), 32.8, 32.7 (C-2′), 20.9, 20.6 (C-3′), 146.7, 146.3 (C-4′), 121.3 (2C, 2×C-5′), 53.5 (2C, 2×CH_2_S), 132.1 (2C, 2×C-1′′), 132.0 (4C, 2×C-2′′ and 2×C-6′′), 129.3 (4C, 2×C-3′′ and 2×C-5′′), 128.7 (2C, 2×C-4′′); EIMS *m/z* 837.3104 [M+H]^+^ (calcd for C_45_H_53_N_6_O_6_S_2_, 837.3468).

*Methyl(11,12-O-(4-(((1-phenylthio)methyl)-1H-1,2,3-triazol-4-yl)-butanoyloxy)-abieta-8,11,13-triene)-20-oate* (**10**). Pale yellow resin; ${\left[\alpha \right]}_{D}^{20}$ +19 (*c* 0.204, CHCl_3_); IR ν_max_ (film) 3148, 2959, 2870, 1769, 1746, 1489, 1112, 760 cm^−1^; ^1^H-NMR (CDCl_3_): δ 7.46, 7.38 (each 1H, s, H-6′), 7.26–7.31 (10H, m, 2×SPh), 6.94 (1H, s, H-14), 5.57 (4H, brs, 2×CH_2_S), 3.46 (3H, s, COOMe), 3.23 (1H, brd, *J =* 12.1 Hz, H-1), 2.87–2.95 (2H, m, H-7), 2.73–2.85 (5H, m, H-15 and 2×H-4′), 2.45–2.63 (4H, m, 2×H-2′), 2.28 (1H, m, H-6), 1.98–2.09 (5H, m, H-2 and 2×H-3′), 1.84 (1H, m, H-6), 1.44–1.53 (3H, m, H-2; H-3 and H-5), 1.20–1.29 (2H, m, H-1 and H-3), 1.18 (3H, d, *J =* 6.9 Hz, H-16), 1.10 (3H, d, *J =* 6.9 Hz, H-17), 0.95 (3H, s, H-18), 0.72 (3H, s, H-19); ^13^C-NMR (CDCl_3_): δ 34.6 (C-1), 19.8 (C-2), 41.0 (C-3), 33.8 (C-4), 53.4 (C-5), 18.2 (C-6), 31.8 (C-7), 136.6 (C-8), 131.6 (C-9), 47.6 (C-10), 141.2 (C-11), 138.4 (C-12), 139.5 (C-13), 124.9 (C-14), 27.2 (C-15), 22.6 (C-16), 22.9 (C-17), 32.4 (C-18), 19.6 (C-19), 175.2 (C-20), 51.6 (OMe), 170.7, 170.4 (C-1′), 32.9, 32.8 (C-2′), 24.4, 23.9 (C-3′), 24.7 (2C, 2×C-4′), 147.7, 147.3 (C-5′), 120.7, 120.6 (C-6′), 53.6, 53.5 (CH_2_S), 132.1 (2C, 2×C-1′′), 132.1 (4C, 2×C-2′′ and 2×C-6′′), 129.3 (4C, 2×C-3′′ and 2×C-5′′), 128.1 (2C, 2×C-4′′); EIMS *m/z* 865.3250 [M+H]^+^ (calcd for C_47_H_57_N_6_O_6_S_2_, 865.3781).

*Methyl(11,12-O-(3-(1-benzyl-1H-1,2,3-triazol-4-yl)-propanoyloxy)-abieta-8,11,13-triene)-20-oate* (**11**). White resin; ${\left[\alpha \right]}_{D}^{20}$ +21 (*c* 0.192, CHCl_3_); IR ν_max_ (film) 3134, 2959, 2867, 1769, 1749, 1437, 1109, 752 cm^−1^; ^1^H-NMR (CDCl_3_): δ 7.43 (2H, brs, 2×H-5′), 7.31–7.34 (6H, m, 2×H-2′′; 2×H-4′′ and 2×H-6′′), 7.22–7.26 (4H, m, 2×H-3′′ and 2×H-5′′), 6.93 (1H, s, H-14), 5.45, 5.42 (each 2H, s, CH_2_Ph), 3.44 (3H, s, COOMe), 3.22 (1H, brd, *J =* 12.5 Hz, H-1), 2.97–3.04 (4H, m, 2×H-3′), 2.89–2.95 (2H, m, H-7), 2.65–2.88 (5H, m, H-15 and 2×H-2′), 2.31 (1H, m, H-6), 2.08 (1H, m, H-2), 1.85 (1H, m, H-6), 1.45–1.53 (3H, m, H-2; H-3 and H-5), 1.21–1.30 (2H, m, H-1 and H-3), 1.15 (3H, d, *J =* 6.9 Hz, H-16), 1.03 (3H, d, *J =* 6.9 Hz, H-17), 0.97 (3H, s, H-18), 0.73 (3H, s, H-19); ^13^C-NMR (CDCl_3_): δ 35.1 (C-1), 20.3 (C-2), 41.5 (C-3), 34.3 (C-4), 54.0 (C-5), 18.7 (C-6), 32.3 (C-7), 135.3 (C-8), 129.4 (C-9), 48.1 (C-10), 141.7 (C-11), 137.1 (C-12), 140.9 (C-13), 125.5 (C-14), 27.6 (C-15), 22.9 (C-16), 23.4 (C-17), 32.8 (C-18), 20.1 (C-19), 175.6 (C-20), 52.1 (OMe), 170.8, 170.5 (C-1′), 33.5, 33.3 (C-2′), 21.2, 21.0 (C-3′), 147.1, 146.7 (C-4′), 122.2 (2C, 2×C-5′), 54.4 (2C, 2×CH_2_Ph), 135.3 (2C, 2×C-1′′), 129.4 (4C, 2×C-2′′ and 2×C-6′′), 128.5 (4C, 2×C-3′′ and 2×C-5′′), 129.0 (2C, 2×C-4′′); EIMS *m/z* 773.3461 [M+H]^+^ (calcd for C_45_H_53_N_6_O_6_, 773.4027).

*Methyl(11,12-O-(4-(1-benzyl-1H-1,2,3-triazol-4-yl)-butanoyloxy)-abieta-8,11,13-triene)-20-oate* (**12**). White resin; ${\left[\alpha \right]}_{D}^{20}$ +24 (*c* 0.187, CHCl_3_); IR ν_max_ (film) 3142, 2959, 2867, 1772, 1746, 1460, 1112, 757 cm^−1^; ^1^H-NMR (CDCl_3_): δ 7.41, 7.31 (each 1H, s, H-6′), 7.31–7.34 (6H, m, 2×H-2′′; 2×H-4′′′ and 2×H-6′′), 7.25–7.28 (4H, m, 2×H-3′′ and 2×H-5′′), 6.95 (1H, s, H-14), 5.48 (4H, brs, 2×CH_2_Ph), 3.46 (3H, s, COOMe), 3.23 (1H, brd, *J =* 12.2 Hz, H-1), 2.88–2.96 (2H, m, H-7), 2.75–2.87 (5H, m, H-15 and 2×H-4′), 2.47–2.66 (4H, m, 2×H-2′), 2.29 (1H, m, H-6), 1.99–2.11 (5H, m, H-2 and 2×H-3′), 1.85 (1H, m, H-6), 1.45–1.54 (3H, m, H-2; H-3 and H-5), 1.20–1.31 (2H, m, H-1 and H-3), 1.20 (3H, d, *J =* 6.9 Hz, H-16), 1.11 (3H, d, *J =* 6.9 Hz, H-17), 0.97 (3H, s, H-18), 0.74 (3H, s, H-19); ^13^C-NMR (CDCl_3_): δ 35.1 (C-1), 20.2 (C-2), 41.5 (C-3), 34.3 (C-4), 53.9 (C-5), 18.7 (C-6), 32.3 (C-7), 135.4 (C-8), 132.1 (C-9), 48.1 (C-10), 141.8 (C-11), 137.0 (C-12), 140.1 (C-13), 125.4 (C-14), 27.7 (C-15), 23.1 (C-16), 23.4 (C-17), 32.9 (C-18), 20.1 (C-19), 175.6 (C-20), 52.1 (OMe), 171.2, 171.0 (C-1′), 33.5, 33.4 (C-2′), 24.9, 24.4 (C-3′), 25.3, 25.2 (C-4′), 148.1, 147.7 (C-5′), 121.6, 121.5 (C-6′), 54.4 (2C, 2×CH_2_Ph), 135.4 (2C, 2×C-1′′), 129.4 (4C, 2×C-2” and 2×C-6′′), 128.4 (4C, 2×C-3′′ and 2×C-5′′), 129.0 (2×C-4′′); EIMS *m/z* 801.3727 [M+H]^+^ (calcd for C_47_H_57_N_6_O_6_, 801.4340).

*Methyl(11,12-O-(3-(1-(4-bromobenzyl)-1H-1,2,3-triazol-4-yl)-propanoyloxy)-abieta-8,11,13-triene)-20-oate* (**13**). White resin; ${\left[\alpha \right]}_{D}^{20}$ +72 (*c* 0.133, CHCl_3_); IR ν_max_ (film) 3145, 2956, 2867, 1801, 1760, 1443, 1131, 757 cm^−1^; ^1^H-NMR (CDCl_3_): δ 7.46 (4H, brd, *J =* 7.7 Hz, 2×H-3′′ and 2×H-5′′), 7.45 (2H, brs, 2×H-5′), 7.13, 7.10 (each 2H, d, *J =* 8.9 Hz, H-2′′ and H-6′′), 6.94 (1H, s, H-14), 5.41, 5.38 (each 2H, brs, CH_2_PhBr), 3.45 (3H, s, COOMe), 3.20 (1H, brd, *J =* 12.3 Hz, H-1), 2.97–3.04 (4H, m, 2×H-3′), 2.88–2.95 (2H, m, H-7), 2.64–2.87 (5H, m, H-15 and 2×H-2′), 2.30 (1H, m, H-6), 2.07 (1H, m, H-2), 1.85 (1H, m, H-6), 1.45–1.53 (3H, m, H-2; H-3 and H-5), 1.20–1.29 (2H, m, H-1 and H-3), 1.15 (3H, d, *J =* 6.9 Hz, H-16), 1.02 (3H, d, *J =* 6.9 Hz, H-17), 0.97 (3H, s, H-18), 0.73 (3H, s, H-19); ^13^C-NMR (CDCl_3_): δ 35.1 (C-1), 20.3 (C-2), 41.5 (C-3), 34.3 (C-4), 54.0 (C-5), 18.7 (C-6), 32.3 (C-7), 136.3 (C-8), 131.3 (C-9), 48.1 (C-10), 141.6 (C-11), 137.2 (C-12), 140.1 (C-13), 125.5 (C-14), 27.6 (C-15), 22.9 (C-16), 23.4 (C-17), 32.8 (C-18), 20.1 (C-19), 175.6 (C-20), 52.1 (OMe), 170.7, 170.5 (C-1′), 33.5, 33.3 (C-2′), 21.2, 20.9 (C-3′), 146.9, 146.5 (C-4′), 122.2 (2C, 2×C-5′), 53.7 (2C, 2×CH_2_PhBr), 134.3 (2C, 2×C-1′′), 130.1 (4C, 2×C-2′′ and 2×C-6′′), 132.6 (4C, 2×C-3′′ and 2×C-5′′), 123.1 (2C, 2×C-4′′); EIMS *m/z* 929.2422 [M+H]^+^ (calcd for C_45_H_51_Br_2_N_6_O_6_, 929.2237).

*Methyl(11,12-O-(4-(1-(4-bromobenzyl)-1H-1,2,3-triazol-4-yl)-butanoyloxy)-abieta-8,11,13-triene)-20-oate* (**14**). White resin; ${\left[\alpha \right]}_{D}^{20}$ +77 (*c* 0.121, CHCl_3_); IR ν_max_ (film) 3142, 2956, 2870, 1801, 1760, 1440, 1128, 757 cm^−1^; ^1^H-NMR (CDCl_3_): δ 7.46 (4H, brd, *J =* 8.3 Hz, 2×H-3′′ and 2×H-5′′), 7.43, 7.34 (each 1H, s, H-6′), 7.13, 7.12 (each 2H, d, *J =* 8.3 Hz, H-2′′ and H-6′′), 6.94 (1H, s, H-14), 5.43 (4H, brs, 2×CH_2_PhBr), 3.45 (3H, s, COOMe), 3.21 (1H, brd, *J =* 12.1 Hz, H-1), 2.85–2.95 (2H, m, H-7), 2.75–2.83 (5H, m, H-15 and 2×H-4′), 2.49–2.65 (4H, m, 2×H-2′), 2.28 (1H, m, H-6), 2.00–2.08 (5H, m, H-2 and 2×H-3′), 1.85 (1H, m, H-6), 1.44–1.53 (3H, m, H-2; H-3 and H-5), 1.20–1.31 (2H, m, H-1 and H-3), 1.17 (3H, d, *J =* 6.9 Hz, H-16), 1.10 (3H, d, *J =* 6.9 Hz, H-17), 0.97 (3H, s, H-18), 0.73 (3H, s, H-19); ^13^C-NMR (CDCl_3_): δ 35.1 (C-1), 20.2 (C-2), 41.4 (C-3), 34.3 (C-4), 53.9 (C-5), 18.7 (C-6), 32.3 (C-7), 137.1 (C-8), 131.4 (C-9), 48.1 (C-10), 141.2 (C-11), 139.0 (C-12), 140.1 (C-13), 125.4 (C-14), 27.7 (C-15), 23.1 (C-16), 23.4 (C-17), 32.9 (C-18), 20.1 (C-19), 175.7 (C-20), 52.1 (OMe), 171.2, 170.9 (C-1′), 33.5, 33.4 (C-2′), 24.9, 24.4 (C-3′), 25.3, 25.2 (C-4′), 148.2, 147.9 (C-5′), 121.6, 121.5 (C-6′), 53.7 (2C, 2×CH_2_PhBr), 134.4 (2C, 2×C-1′′), 130.1 (4C, 2×C-2′′ and 2×C-6′′), 132.6 (4C, 2×C-3′′ and 2×C-5′′), 123.1 (2C, 2×C-4′′); EIMS *m/z* 957.2837 [M+H]^+^ (calcd for C_47_H_55_Br_2_N_6_O_6_, 957.2550).

*Methyl(11,12-O-(3-(1-(4-nitrobenzyl)-1H-1,2,3-triazol-4-yl)-propanoyloxy)-abieta-8,11,13-triene)-20-oate* (**15**). Colorless resin; ${\left[\alpha \right]}_{D}^{20}$ +89 (*c* 0.167, CHCl_3_); IR ν_max_ (film) 3142, 2956, 2867, 1801, 1760, 1437, 1131, 754 cm^−1^; ^1^H-NMR (CDCl_3_): δ 8.20, 8.19 (each 2H, d, *J =* 8.3 Hz, H-3′′ and H-5′′), 7.52, 7.50 (each 1H, s, H-5′), 7.40 (4H, brd, *J =* 8.5 Hz, 2×H-2′′ and 2×H-6′′), 6.94 (1H, s, H-14), 5.60 (4H, brs, 2×CH_2_PhNO_2_), 3.46 (3H, s, COOMe), 3.21 (1H, brd, *J =* 12.7 Hz, H-1), 3.00–3.07 (4H, m, 2×H-3′), 2.91–2.98 (2H, m, H-7), 2.70–2.90 (5H, m, H-15 and 2×H-2′), 2.31 (1H, m, H-6), 2.07 (1H, m, H-2), 1.85 (1H, m, H-6), 1.46–1.53 (3H, m, H-2; H-3 and H-5), 1.21–1.29 (2H, m, H-1 and H-3), 1.15 (3H, d, *J =* 6.9 Hz, H-16), 1.03 (3H, d , *J =* 6.9 Hz, H-17), 0.98 (3H, s, H-18), 0.74 (3H, s, H-19); ^13^C-NMR (CDCl_3_): δ 35.2 (C-1), 20.3 (C-2), 41.5 (C-3), 34.4 (C-4), 54.0 (C-5), 18.7 (C-6), 32.2 (C-7), 137.4 (C-8), 132.1 (C-9), 48.1 (C-10), 141.5 (C-11), 138.8 (C-12), 140.1 (C-13), 125.6 (C-14), 27.7 (C-15), 22.9 (C-16), 23.4 (C-17), 32.8 (C-18), 20.1 (C-19), 175.7 (C-20), 52.1 (OMe), 170.7, 170.5 (C-1′), 33.5, 33.3 (C-2′), 21.2, 20.9 (C-3′), 147.6, 147.1 (C-4′), 122.6, 122.5 (C-5′), 53.4 (2C, 2×CH_2_PhNO_2_), 142.3, 142.2 (C-1′′), 129.0 (4C, 2×C-2′′ and 2×C-6′′), 124.6 (4C, 2×C-3′′ and 2×C-5′′), 148.4 (2C, 2×C-4′′); EIMS *m/z* 863.4050 [M+H]^+^ (calcd for C_45_H_51_N_8_O_10_, 863.3728).

*Methyl(11,12-O-(4-(1-(4-nitrobenzyl)-1H-1,2,3-triazol-4-yl)-butanoyloxy)-abieta-8,11,13-triene)-20-oate* (**16**). Colorless resin; ${\left[\alpha \right]}_{D}^{20}$ +64 (*c* 0.131, CHCl_3_); IR ν_max_ (film) 3139, 2956, 2867, 1772, 1718, 1454, 1120, 754 cm^−1^; ^1^H-NMR (CDCl_3_): δ 8.18, 8.17 (each 2H, d, *J =* 8.5 Hz, H-3′′ and H-5′′), 7.52, 7.43 (each 1H, s, H-6′), 7.40, 7.39 (each 2H, d, *J =* 8.5 Hz, H-2′′ and H-6′′), 6.94 (1H, s, H-14), 5.62 (4H, brs, 2×CH_2_PhNO_2_), 3.44 (3H, s, COOMe), 3.20 (1H, brd, *J =* 12.2 Hz, H-1), 2.85–2.95 (2H, m, H-7), 2.78–2.84 (5H, m, H-15 and 2×H-4′), 2.49–2.66 (4H, m, 2×H-2′), 2.27 (1H, m, H-6), 2.00–2.10 (5H, m, H-2 and 2×H-3′), 1.85 (1H, m, H-6), 1.42–1.53 (3H, m, H-2; H-3 and H-5), 1.19–1.30 (2H, m, H-1 and H-3), 1.16 (3H, d, *J =* 6.9 Hz, H-17), 1.09 (3H, d, *J =* 6.9 Hz, H-16), 0.96 (3H, s, H-18), 0.71 (3H, s, H-19); ^13^C-NMR (CDCl_3_): δ 35.1 (C-1), 20.2 (C-2), 41.4 (C-3), 34.3 (C-4), 53.9 (C-5), 18.7 (C-6), 32.2 (C-7), 137.2 (C-8), 132.1 (C-9), 48.1 (C-10), 141.7 (C-11), 138.9 (C-12), 140.1 (C-13), 125.5 (C-14), 27.7 (C-15), 23.1 (C-16), 23.4 (C-17), 32.8 (C-18), 20.1 (C-19), 175.7 (C-20), 52.1 (OMe), 171.2, 170.1 (C-1′), 33.5, 33.4 (C-2′), 24.9, 24.3 (C-3′), 25.3, 25.2 (C-4′), 148.1, 147.8 (C-5′), 122.0, 121.9 (C-6′), 53.3 (2C, 2×CH_2_PhNO_2_), 142.5 (2C, 2×C-1′′), 129.0 (4C, 2×C-2′′ and 2×C-6′′), 124.6 (4C, 2×C-3′′ and 2×C-5′′), 148.4 (2C, 2×C-4′′); EIMS *m/z* 891.4382 [M+H]^+^ (calcd for C_47_H_55_N_8_O_10_, 891.4041).

*(7β)-11,12-O-(3-(((1-phenylthio)methyl)-1H-1,2,3-triazol-4-yl)-propanoyloxy)-7,20-epoxyabieta-8,11,13-trien-20-one* (**17**). Pale yellow resin; ${\left[\alpha \right]}_{D}^{20}$ +61 (*c* 0.162, CHCl_3_); IR ν_max_ (film) 3133, 2959, 2870, 1769, 1715, 1460, 1120, 757 cm^−1^; ^1^H-NMR (CDCl_3_): δ 7.49, 7.45 (each 1H, s, H-5′), 7.25-7.33 (10H, m, 2×SPh), 7.08 (1H, s, H-14), 5.63, 5.57 (each 2H, brs, CH_2_S), 5.47 (1H, d, *J =* 2.7 Hz, H-7α), 3.04 (4H, m, 2×H-3′), 2.88–2.96 (4H, m, 2×H-2′), 2.80–2.87 (1H, m, H-15), 2.22 (1H, m, H-6β), 1.88–2.00 (1H, m, H-1α), 184–190 (2H, m, H-2α and H-6α), 1.72–1.82 (1H, m, H-1β), 1.60 (1H, dd, *J =* 10.5, 5.8 Hz, H-5), 1.44–1.48 (2H, m, H-2β and H-3α), 1.09–1.15 (1H, m, H-3β), 1.13 (3H, d, *J =* 7.5 Hz, H-16), 1.11 (3H, d, *J =* 7.5 Hz, H-17), 0.86 (3H, s, H-18), 0.81 (3H, s, H-19); ^13^C-NMR (CDCl_3_): δ 28.4 (C-1), 19.1 (C-2), 41.0 (C-3), 34.9 (C-4), 44.9 (C-5), 29.5 (C-6), 77.5 (C-7), 129.6 (C-8), 138.5 (C-9), 48.4 (C-10), 141.2 (C-11), 141.7 (C-12), 139.4 (C-13), 119.0 (C-14), 27.9 (C-15), 23.2 (C-16), 23.3 (C-17), 20.1 (C-18), 32.0 (C-19), 174.6 (C-20), 170.8, 170.7 (C-1′), 33.8, 33.1 (C-2′), 21.1, 20.9 (C-3′), 146.6, 146.5 (C-4′), 121.8 (2C, 2×C-5′), 54.2, 54.1 (CH_2_S), 132.5 (2C, 2×C-1′′), 132.6 (4C, 2×C-2′′ and 2×C-6′′), 129.9, 129.8 (each 2C, C-3′′ and C-5′′), 129.1, 128.9 (C-4′′); EIMS *m/z* 821.3427 [M+H]^+^ (calcd for C_44_H_49_N_6_O_6_S_2_, 821.3155).

*(7β)-11,12-O-(4-(((1-phenylthio)methyl)-1H-1,2,3-triazol-4-yl)-butanoyloxy)-7,20-epoxyabieta-8,11,13-trien-20-one* (**18**). Pale yellow resin; ${\left[\alpha \right]}_{D}^{20}$ +56 (*c* 0.153, CHCl_3_); IR ν_max_ (film) 3132, 2957, 2869, 1771, 1718, 1456, 1120, 756 cm^−1^; ^1^H-NMR (CDCl_3_): δ 7.37, 7.36 (each 1H, s, H-6′), 7.25–7.30 (10H, m, 2×SPh), 7.07 (1H, s, H-14), 5.57, 5.56 (each 2H, brs, CH_2_S), 5.46 (1H, brs, H-7α), 2.85 (1H, m, H-15), 2.74 (4H, m, 2×H-4′), 2.55 (4H, m, 2×H-2′), 2.22 (1H, m, H-6β), 1.93–2.07 (5H, m, H-1α and 2×H-3′), 185–191 (2H, m, H-2α and H-6α), 1.71–1.76 (1H, m, H-1β), 1.67 (1H, dd, *J =* 10.1, 5.5 Hz, H-5), 1.46–1.54 (2H, m, H-2β and H-3α), 1.13–1.24 (1H, m, H-3β), 1.13 (6H, brd, *J =* 6.6 Hz, H-16 and H-17), 0.86 (3H, s, H-18), 0.82 (3H, s, H-19); ^13^C-NMR (CDCl_3_): δ 28.0 (C-1), 18.6 (C-2), 40.5 (C-3), 34.4 (C-4), 44.4 (C-5), 29.0 (C-6), 77.0 (C-7), 130.8 (C-8), 137.9 (C-9), 48.3 (C-10), 140.7 (C-11), 141.1 (C-12), 139.0 (C-13), 118.4 (C-14), 27.4 (C-15), 23.9 (C-16), 24.3 (C-17), 20.1 (C-18), 31.5 (C-19), 174.1 (C-20), 170.7, 170.6 (C-1′), 32.9, 32.8 (C-2′), 22.8, 22.7 (C-3′), 24.6 (2C, 2×C-4′), 147.1 (2C, 2×C-5′), 120.6 (2C, 2×C-6′), 53.5 (2C, 2×CH_2_S), 131.9 (2C, 2×C-1′′), 132.0 (4C, 2×C-2′′ and 2×C-6′′), 129.3 (4C, 2×C-3′′ and 2×C-5′′), 128.5 (2C, 2×C-4′′); EIMS *m/z* 849.3661 [M+H]^+^ (calcd for C_46_H_53_N_6_O_6_S_2_, 849.3468). 

*(7β)-11,12-O-(3-(1-benzyl-1H-1,2,3-triazol-4-yl)-propanoyloxy)-7,20-epoxyabieta-8,11,13-trien-20-one* (**19**). Colorless resin; ${\left[\alpha \right]}_{D}^{20}$ +67 (*c* 0.039, CHCl_3_); IR ν_max_ (film) 3140, 2959, 2871, 1769, 1716, 1460, 1121, 756 cm^−1^; ^1^H-NMR (CDCl_3_): δ 7.68, 7.50 (each 1H, s, H-5′), 7.27–7.34 (6H, m, 2×H-2′′; 2×H-4′′ and 2×H-6′′), 7.19–7.27 (4H, m, 2×H-3′′ and 2×H-5′′), 7.06 (1H, s, H-14), 5.44 (5H, brs, H-7α and 2×CH_2_S), 2.96–3.14 (4H, m, 2×H-3′), 2.84–2.96 (4H, m, 2×H-2′), 2.75–2.84 (1H, m, H-15), 2.21 (1H, m, H-6β), 1.82–1.93 (3H, m, H-1α; H-2α and H-6α), 1.68–1.76 (1H, m, H-1β), 1.63 (1H, dd, *J =* 10.5, 5.8 Hz, H-5), 1.45–1.50 (2H, m, H-2β and H-3α), 1.07–1.13 (1H, m, H-3β), 1.09 (6H, brd, *J =* 6.6 Hz, H-16 and H-17), 0.86 (3H, s, H-18), 0.82 (3H, s, H-19); ^13^C-NMR (CDCl_3_): δ 28.4 (C-1), 19.1 (C-2), 41.1 (C-3), 35.0 (C-4), 44.9 (C-5), 29.6 (C-6), 77.6 (C-7), 129.2 (C-8), 138.5 (C-9), 48.7 (C-10), 141.2 (C-11), 141.7 (C-12), 139.4 (C-13), 119.1 (C-14), 27.9 (C-15), 23.2 (C-16), 23.3 (C-17), 20.1 (C-18), 32.0 (C-19), 174.6 (C-20), 170.9, 170.8 (C-1′), 33.6, 33.4 (C-2′), 21.4, 21.0 (C-3′), 147.1, 147.9 (C-4′), 121.9 (2C, 2×C-5′), 54.9 (2C, 2×CH_2_Ph), 135.3, 135.1 (C-1′′), 129.5, 129.4 (each 2C, C-2′′ and C-6′′), 128.7, 128.6 (each 2C, C-3′′ and C-5′′), 129.1, 129.0 (C-4′′); EIMS *m/z* 757.4006 [M+H]^+^ (calcd for C_44_H_49_N_6_O_6_, 757.3741). 

*(7β)-11,12-O-(4-(1-benzyl-1H-1,2,3-triazol-4-yl)-butanoyloxy)-7,20-epoxyabieta-8,11,13-trien-20-one* (**20**). Colorless resin; ${\left[\alpha \right]}_{D}^{20}$ +49 (*c* 0.039, CHCl_3_); IR ν_max_ (film) 3142, 2959, 2870, 1767, 1718, 1460, 1120, 754 cm^−1^; ^1^H-NMR (CDCl_3_): δ 7.32–7.36 (8H, m, 2×H-6′; 2×H-2′′; 2×H-4′′ and 2×H-6′′), 7.25–7.28 (4H, m, 2×H-3′′ and 2×H-5′′), 7.09 (1H, s, H-14), 5.48 (5H, brs, H-7α and 2×CH_2_S), 2.88 (1H, m, H-15), 2.78 (4H, m, 2×H-4′), 2.60 (4H, m, 2×H-2′), 2.23 (1H, m, H-6β), 2.03–2.12 (5H, m, H-1α and 2×H-3′), 188–194 (2H, m, H-2α and H-6α), 1.72–1.78 (1H, m, H-1β), 1.69 (1H, dd, *J =* 10.5, 5.5 Hz, H-5), 1.49–1.55 (2H, m, H-2β and H-3α), 1.14–1.25 (1H, m, H-3β), 1.15 (6H, brd, *J =* 6.7 Hz, H-16 and H-17), 0.90 (3H, s, H-18), 0.85 (3H, s, H-19); ^13^C-NMR (CDCl_3_): δ 28.4 (C-1), 19.1 (C-2), 41.1 (C-3), 35.0 (C-4), 45.0 (C-5), 29.6 (C-6), 77.6 (C-7), 129.5 (C-8), 138.4 (C-9), 48.1 (C-10), 141.2 (C-11), 141.7 (C-12), 139.4 (C-13), 118.9 (C-14), 28.0 (C-15), 23.2 (C-16), 23.3 (C-17), 20.1 (C-18), 32.0 (C-19), 174.6 (C-20), 170.9, 170.8 (C-1′), 33.6, 33.4 (C-2′), 24.9, 24.5 (C-3′), 25.2 (2C, 2×C-4′), 147.6, 147.4 (C-5′), 121.5 (2C, 2×C-6′), 54.4 (2C, 2×CH_2_Ph), 135.3 (2C, 2×C-1′′), 129.5 (4C, 2×C-2′′ and 2×C-6′′), 128.4 (4C, 2×C-3′′ and 2×C-5′′), 129.0 (2C, 2×C-4′′); EIMS *m/z* 785.4452 [M+H]^+^ (calcd for C_46_H_53_N_6_O_6_, 785.4027).

*(7β)-11,12-O-(3-(1-(4-bromobenzyl)-1H-1,2,3-triazol-4-yl)-propanoyloxy)-7,20-epoxyabieta-8,11,13-trien-20-one* (**21**). White resin; ${\left[\alpha \right]}_{D}^{20}$ +53 (*c* 0.140, CHCl_3_); IR ν_max_ (film) 3136, 2956, 2867, 1763, 1715, 1454, 1123, 757 cm^−1^; ^1^H-NMR (CDCl_3_): δ 7.47, 7.46 (each 2H, d, *J =* 8.3 Hz, H-3′′ and H-5′′), 7.42, 7.40 (each 1H, s, H-5′), 7.14, 7.13 (each 2H, d, *J =* 8.1 Hz, H-2′′ and H-6′′), 7.08 (1H, s, H-14), 5.48 (1H, d, *J =* 2.7 Hz, H-7α), 5.46, 5.42 (each 2H, brs, CH_2_PhBr), 3.05 (4H, m, 2×H-3′), 2.85–2.90 (4H, m, 2×H-2′), 2.78–2.85 (1H, m, H-15), 2.23 (1H, m, H-6β), 1.88–2.00 (1H, m, H-1α), 185–191 (2H, m, H-2α and H-6α), 1.74–1.80 (1H, m, H-1β), 1.65 (1H, dd, *J =* 10.6, 5.8 Hz, H-5), 1.46–1.50 (2H, m, H-2β and H-3α), 1.09–1.15 (1H, m, H-3β), 1.13 (3H, d, *J =* 6.8 Hz, H-16), 1.09 (3H, d, *J =* 6.8 Hz, H-17), 0.88 (3H, s, H-18), 0.84 (3H, s, H-19); ^13^C-NMR (CDCl_3_): δ 28.3 (C-1), 19.1 (C-2), 41.0 (C-3), 35.0 (C-4), 44.9 (C-5), 29.5 (C-6), 77.5 (C-7), 129.6 (C-8), 138.5 (C-9), 48.7 (C-10), 141.1 (C-11), 141.7 (C-12), 139.4 (C-13), 119.1 (C-14), 27.9 (C-15), 23.2 (2C, C-16 and C-17), 20.1 (C-18), 32.0 (C-19), 174.6 (C-20), 170.9, 170.7 (C-1′), 33.1 (2C, 2×C-2′), 21.1, 20.9 (C-3′), 147.0, 146.8 (C-4′), 122.2 (2C, 2×C-5′), 53.7 (2C, 2×CH_2_PhBr), 134.3, 134.2 (C-1′′), 130.2, 130.1 (each 2C, C-2′′ and C-6′′), 132.6, 132.6 (each 2C, C-3′′ and C-5′′), 123.2, 123.1 (C-4′′); EIMS *m/z* 913.1844 [M+H]^+^ (calcd for C_44_H_47_Br_2_N_6_O_6_, 913.1924).

*(7β)-11,12-O-(4-(1-(4-bromobenzyl)-1H-1,2,3-triazol-4-yl)-butanoyloxy)-7,20-epoxyabieta-8,11,13-trien-20-one* (**22**). White resin; ${\left[\alpha \right]}_{D}^{20}$ +55 (*c* 0.132, CHCl_3_); IR ν_max_ (film) 3133, 2962, 2873, 1769, 1752, 1454, 1109, 754 cm^−1^; ^1^H-NMR (CDCl_3_): δ 7.45 (4H, d, *J =* 8.3 Hz, H-3′′ and H-5′′), 7.35 (2H, s, 2×H-6′), 7.13 (4H, brd, *J =* 8.3 Hz, H-2′′ and H-6′′), 7.09 (1H, s, H-14), 5.48 (1H, d, *J =* 2.4 Hz, H-7α), 5.43 (4H, brs, 2×CH_2_PhBr), 2.87 (1H, m, H-15), 2.78 (4H, m, 2×H-4′), 2.60 (4H, m, 2×H-2′), 2.22 (1H, m, H-6β), 2.03–2.10 (5H, m, H-1α and 2×H-3′), 188–194 (2H, m, H-2α and H-6α), 1.72–1.76 (1H, m, H-1β), 1.67 (1H, dd, *J =* 10.4, 5.8 Hz, H-5), 1.48–1.53 (2H, m, H-2β and H-3α), 1.14–1.24 (1H, m, H-3β), 1.14 (6H, brd, *J =* 6.8 Hz, H-16 and H-17), 0.88 (3H, s, H-18), 0.84 (3H, s, H-19); ^13^C-NMR (CDCl_3_): δ 28.5 (C-1), 19.1 (C-2), 41.1 (C-3), 35.0 (C-4), 45.0 (C-5), 29.5 (C-6), 77.5 (C-7), 129.4 (C-8), 138.5 (C-9), 48.1 (C-10), 141.1 (C-11), 141.7 (C-12), 139.5 (C-13), 19.0 (C-14), 28.0 (C-15), 23.2 (C-16), 23.3 (C-17), 20.1 (C-18), 32.0 (C-19), 174.6 (C-20), 171.2, 170.9 (C-1′), 33.4 (2C, 2×C-2′), 24.8, 24.5 (C-3′), 25.2 (2C, 2×C-4′), 147.7 (2C, 2×C-5′), 121.5 (2C, 2×C-6′), 53.7 (2C, 2×CH_2_PhBr), 134.4 (2C, 2×C-1′′), 130.1 (4C, 2×C-2′′ and 2×C-6′′), 132.6 (4C, 2×C-3′′ and 2×C-5′′), 123.1 (2C, 2×C-4′′); EIMS *m/z* 941.2499 [M+H]^+^ (calcd for C_46_H_51_Br_2_N_6_O_6_, 941.2237).

*(7β)-11,12-O-(3-(1-(4-nitrobenzyl)-1H-1,2,3-triazol-4-yl)-propanoyloxy)-7,20-epoxyabieta-8,11,13-trien-20-one* (**23**). White resin; ${\left[\alpha \right]}_{D}^{20}$ +18 (*c* 0.193, CHCl_3_); IR ν_max_ (film) 3136, 2957, 2868, 1765, 1744, 1458, 1111, 757 cm^−1^; ^1^H-NMR (CDCl_3_): δ 8.17, 8.16 (each 2H, d, *J =* 8.6 Hz, H-3′′ and H-5′′), 7.49, 7.46 (each 1H, s, H-5′), 7.41, 7.38 (each 2H, d, *J =* 8.6 Hz, 2×H-2′′ and 2×H-6′′), 7.09 (1H, s, H-14), 5.66, 5.61 (each 2H, s, CH_2_PhNO_2_), 5.49 (1H, d, *J =* 2.7 Hz, H-7α), 3.06 (4H, m, 2×H-3′), 2.89–2.97 (4H, m, 2×H-2′), 2.79–2.89 (1H, m, H-15), 2.23 (1H, m, H-6β), 1.99–2.10 (1H, m, H-1α), 187–194 (2H, m, H-2α and H-6α), 1.74–1.80 (1H, m, H-1β), 1.64 (1H, dd, *J =* 10.6, 5.8 Hz, H-5), 1.44–1.49 (2H, m, H-2β and H-3α), 1.07–1.17 (1H, m, H-3β), 1.12 (3H, d, *J =* 6.9 Hz, H-16), 1.08 (3H, d, *J =* 6.9 Hz, H-17), 0.86 (3H, s, H-18), 0.83 (3H, s, H-19); ^13^C-NMR (CDCl_3_): δ 28.4 (C-1), 19.1 (C-2), 40.9 (C-3), 34.9 (C-4), 44.9 (C-5), 29.5 (C-6), 77.5 (C-7), 129.6 (C-8), 138.6 (C-9), 48.7 (C-10), 141.1 (C-11), 141.8 (C-12), 139.3 (C-13), 119.2 (C-14), 27.9 (C-15), 23.2 (2C, C-16 and C-17), 20.1 (C-18), 32.0 (C-19), 174.7 (C-20), 171.0, 170.7 (C-1′), 33.9, 33.1 (C-2′), 21.0, 20.9 (C-3′), 146.9, 146.8 (C-4′), 122.5 (2C, 2×C-5′), 53.4 (2C, 2×CH_2_PhNO_2_), 142.4, 142.3 (C-1′′), 129.2, 129.0 (each 2C, C-2′′ and C-6′′), 124.6 (4C, 2×C-3′′ and 2×C-5′′), 148.4, 148.3 (C-4′′); EIMS *m/z* 847.3828 [M+H]^+^ (calcd for C_44_H_47_N_8_O_10_, 847.3415). 

*(7β)-11,12-O-(4-(1-(4-nitrobenzyl)-1H-1,2,3-triazol-4-yl)-butanoyloxy)-7,20-epoxyabieta-8,11,13-trien-20-one* (**24**). White resin; ${\left[\alpha \right]}_{D}^{20}$ +26(*c* 0.039, CHCl_3_); IR ν_max_ (film) 3139, 2959, 2870, 1763, 1746, 1457, 1111, 757 cm^−1^; ^1^H-NMR (CDCl_3_): δ 8.16 (4H, brd, *J =* 8.4 Hz, H-3′′ and H-5′′), 7.45, 7.44 (each 1H, s, H-6′), 7.39 (4H, brd, *J =* 7.9 Hz, 2×H-2′′ and 2×H-6′′), 7.10 (1H, s, H-14), 5.62, 5.61 (each 2H, s, CH_2_PhNO_2_), 5.49 (1H, d, *J =* 2.4 Hz, H-7α), 2.87 (1H, m, H-15), 2.81 (4H, m, 2×H-4′), 2.62 (4H, m, 2×H-2′), 2.23 (1H, m, H-6β), 2.03–2.11 (5H, m, H-1α and 2×H-3′), 188–194 (2H, m, H-2α and H-6α), 1.73–1.77 (1H, m, H-1β), 1.67 (1H, dd, *J =* 10.5, 5.7 Hz, H-5), 1.48–1.54 (2H, m, H-2β and H-3α), 1.14–1.23 (1H, m, H-3β), 1.14 (6H, brd, *J =* 6.8 Hz, H-16 and H-17), 0.87 (3H, s, H-18), 0.84 (3H, s, H-19); ^13^C-NMR (CDCl_3_): δ 28.6 (C-1), 19.2 (C-2), 41.1 (C-3), 35.0 (C-4), 45.1 (C-5), 29.6 (C-6), 77.6 (C-7), 129.5 (C-8), 138.5 (C-9), 48.9 (C-10), 141.2 (C-11), 141.8 (C-12), 139.6 (C-13), 119.1 (C-14), 28.1 (C-15), 23.3 (2C, C-16 and C-17), 20.2 (C-18), 32.0 (C-19), 174.8 (C-20), 171.3, 171.2 (C-1′), 33.6, 33.4 (C-2′), 24.9, 24.5 (C-3′), 25.3, 25.2 (C-4′), 147.6 (2C, 2×C-5′), 122.0 (2C, 2×C-6′), 53.4 (2C, 2×CH_2_PhNO_2_), 142.5 (2C, 2×C-1′′), 129.1 (4C, 2×C-2′′ and 2×C-6′′), 124.6 (4C, 2×C-3′′ and 2×C-5′′), 148.4, 148.1 (C-4′′); EIMS *m/z* 875.3885 [M+H]^+^ (calcd for C_46_H_51_N_8_O_10_, 875.3728).

### 3.3. Antiproliferative Assay

All human cell lines used in this work were purchased from the American Type Culture Collection (ATCC, Manasas, VA, USA). Normal lung MRC-5 fibroblasts (CCL-171), SK-MES-1 lung cancer cells (HTB-58) and J82 bladder carcinoma cells (HTB-1) were grown as monolayers in minimum essential Eagle medium (MEM) with Earles’s salts, 2 mM l-glutamine and 1.5 g∙L^−1^ sodium bicarbonate. Gastric adenocarcinoma AGS cells (CRL-1739) were grown as monolayers in Ham F-12 medium containing 1 mM l-glutamine and 1.5 g∙L^−1^ sodium bicarbonate. All media were supplemented with 10% heat-inactivated FBS, 100 IU∙mL^−1^ penicillin and 100 µg∙mL^−1^ streptomycin. Cells were grown in a humidified incubator with 5% CO_2_ in air at 37 °C. For the antiproliferative assay, cells were plated at a density of 5 × 10^4^ cells∙mL^−1^. Cells were seeded in 96-well plates (100 µL∙well^−1^). One day after seeding, cells were treated with medium containing the compounds at concentrations ranging from 0 up to 100 µM during 3 days. The compounds were dissolved in DMSO (1% final concentration) and complete medium. Untreated cells (medium containing 1% DMSO) were used as 100% viability controls. Etoposide (98% purity, Sigma-Aldrich) was used as reference compound. Each concentration was tested in sextuplicate and experiments were repeated 2 times. Cell viability was determined by means of the MTT reduction assay at the end of the incubation with the products. The results were transformed to percentage of controls and the IC_50_ value was obtained adjusting the dose-response curve to a sigmoidal model. The software used was OriginPro 8.1 [30].

### 3.4. Antifungal Evaluation 

#### 3.4.1. Microorganisms and Media

For the antifungal evaluation, standardized strains from the American Type Culture Collection (ATCC), Rockville, MD, USA, were used. The microorganisms included yeasts (*Candida albicans* ATCC 10231 and *Cryptococcus neoformans* ATCC 32264). Strains were grown on Sabouraud-chloramphenicol agar slants for 48 h at 30 °C, maintained on slopes of Sabouraud-dextrose agar (SDA, Oxoid), and subcultured every 15 days to prevent pleomorphic transformations. Inocula of cell or spore suspensions were obtained according to reported procedures and adjusted to 1–5 × 10^3^ cells/spores with colony forming units (CFU)/mL [29].

#### 3.4.2. Antifungal Susceptibility Testing. Fungal Growth Inhibition Percentage Determination

Broth microdilution techniques were performed in 96-well microplates according to the Clinical and Laboratory Standards Institute Reference Method for Broth Dilution Antifungal Susceptibility Testing of Yeasts, Approved Standard M27-A3 [29]. For the assay, compound test wells (CTWs) were prepared with stock solutions of each compound in DMSO (maximum concentration ≤1%), diluted with RPMI-1640, to final concentrations of 250–3.9 μg∙mL^−1^. An inoculum suspension (100 μL) was added to each well (final volume in the well = 200 μL). A growth control well (GCW) (containing medium, inoculum, and the same amount of DMSO used in a CTW, but compound-free) and a sterility control well (SCW) (sample, medium, and sterile water instead of inoculum) were included for each fungus tested. Microtiter trays were incubated in a moist, dark chamber at 30 °C for 48 h for both yeasts. Microplates were read in a VERSA Max microplate reader (Molecular Devices, Sunnyvale, CA, USA). Amphotericin B (Sigma-Aldrich) was used as positive control. Tests were performed in triplicate. Reduction of growth for each compound concentration was calculated as follows: % of inhibition = 100 − (OD 405 CTW − OD 405 SCW)/(OD 405 GCW − OD 405 SCW). The means ± SEM were the results of triplicate tests. Three endpoints were defined from the assay explained above and the dose-response curves. Minimum Inhibitory concentration (MIC) resulting in total fungal growth inhibition was named MIC_100_ while MIC_50_ was defined as the minimum concentration that inhibits 50% of the fungal growth.

## 4. Conclusions 

A series of twenty four novel abietane diterpenes derivatives were synthesized in good to reasonable yields using click chemistry. Modifications were made from three core principles: carnosic acid γ-lactone (CAL), carnosic acid methyl ester (CAM) and carnosol (C). The CAL was attached to a triazole ring while CAM and C were associated to two triazoles. The length of the linker between the terpenes and the triazole was variable (two or three CH_2_ units) and different aromatic rings were present in the triazole moiety. The compounds were assessed for antiproliferative and antifungal properties. The antiproliferative activity was evaluated in three human tumor cell lines and on normal fibroblasts. The formation of the CAL generated compounds with better antiproliferative activity. The antifungal activity of the compounds was determined as percentages of inhibition of *C. albicans* ATCC 10231 and *C. neoformans* ATCC 32264 in the range 250–3.9 µg∙mL^−1^ and from these data, MIC_100_ and MIC_50_ were determined for all compounds. None of the compounds was able to inhibit 100% of fungal growth at 250 μg∙mL^−1^. However, varied percentages of inhibition were displayed by all members of the series at the different tested concentrations. Of both fungi, *C. neoformans* was the most sensitive one, with nine compounds inhibiting more than 50% of its fungal growth at concentrations lower than 250 μg∙mL^−1^. Compound **22** showed the best activity with 91% inhibition growth at 250 μg∙mL^−1^. In turn, six compounds inhibited 50% *C. albicans* growth at concentrations lower than 250 μg∙mL^−1^. These results show the potentiality of carnosic acid and carnosol derivatives for the development of new antiproliferative and antifungal agents.
